# Lessons learnt from the implementation of electronic consent (eConsent) and its use across a large portfolio of trials in a UK academic clinical trials unit

**DOI:** 10.1186/s13063-026-09445-5

**Published:** 2026-01-23

**Authors:** D. E. Appelbe, L. Eldridge, V. S. Barber

**Affiliations:** 1 Oxford Trauma and Emergency Care, Department of Orthopaedics, Rheumatology and Musculoskeletal Sciences, Oxford University, Oxford, UK; 2 Oxford Clinical Trials Research Unit (OCTRU), Department of Orthopaedics, Rheumatology and Musculoskeletal Sciences, Oxford University, Oxford, UK

## Abstract

**Background:**

The use of electronic consent (eConsent) in clinical research studies is on the increase and has been since 2019. eConsent itself can be multi-faceted, encompassing documentation of the consent process, delivering information to the potential participant via the use of different media, and even facilitating a check of the participants’ understanding of the research before they enter a study. Some researchers and research teams have embraced the use of the different aspects of eConsent, whilst other groups/teams are hesitant to employ the methodology until it has been proven and fits better with workflows and patient pathways.

**Main text:**

We report on how eConsent has been utilised in 35 studies from a large academic clinical trials unit in the UK, with nearly 12,000 participants being consented using this methodology via the REDCap data collection system over a 6-year period. The studies utilising eConsent have all made use of the documentation facet of eConsent, with some studies also making use of different media to provide information to the potential participant. The use of these methods has been adopted by the majority of our open studies and all but one of the research delivery teams across the whole CTU portfolio. To facilitate some of the processes that need to be implemented, two external modules have been written for REDCap to make the process easier. Many of our studies also make use of the facility to enable remote eConsent (i.e. where the potential participant and researcher are not physically in the same place), and very few studies in our portfolio now require paper consent forms as either an alternative option or as a backup in case the electronic systems are not available. This has resulted in a reduction in the amount of paper that needs to be provided, resulting in a decrease in the carbon footprint of the studies.

**Conclusions:**

We have successfully utilised eConsent techniques to receive informed consent from nearly 12,000 participants in a multitude of clinical trials recruiting individuals from age 8 to 80. Half of these trials also used eConsent to facilitate both the documentation and the discussion around the consent process. Where remote eConsent has been used, techniques have been employed to make the process easier on the participant and the researcher, whilst not detracting from the two-way conversation that is important for the consent process.

**Supplementary Information:**

The online version contains supplementary material available at 10.1186/s13063-026-09445-5.

## Background

Informed consent is a basic tenant of clinical trials, with the expectations around the process being defined in the 1964 Declaration of Helsinki [1] and the “The Medicines for Human Use (Clinical Trials) Regulations 2004 (as amended)” [2]. The consent process ensures that all potential participants understand “What will happen to them if they take part in research”, “that the research is ethical”, and “that it complies with appropriate legislation” [3]. Regardless of how consent is sought, it is important and good practice for the potential participant to be provided with all pertinent information [4], in an appropriate medium, and that any decision/agreement to participate in a study/trial is documented appropriately.

One mechanism that can be used to deliver and document consent is known as electronic consent (eConsent). eConsent has been defined by the United Kingdom’s (UK) competent authority — the Medicines and Healthcare Products Regulatory Agency (MHRA) as “The use of any electronic media (such as text, graphics, audio, video, podcasts or websites) to convey information related to the study and to seek and/or document informed consent via an electronic device such as a smartphone, tablet or computer” [5]. In a review by Grady et al. [6], they eloquently describe in tabular form the opportunities and challenges of eConsent versus paper, but the challenges and opportunities, which include participant interaction/understanding and national legislation, do not seem to be deterring trialists in its use. How eConsent is utilised by trialists will vary from study to study and population to population, but there is a growing place for it in delivering clinical trials for researchers and patients. A recent publication [7] from the “All of Us” research programme in the US has reported that they were able to eConsent a staggering 705,719 participants from a wide range of backgrounds in a 6-year period through their developed e-consent functionality. Furthermore, a recent review of eConsent in academic clinical trials units (CTU’s) in the UK [8] showed that this tool was being used in clinical studies within the UK, predominantly to record that the consent discussion had taken place. However, in the space of a few years, the number of CTU’s reporting use of online explainer animations to aid in the consent process has also increased [9], perhaps reflecting the changing nature in the general population in how they wish to see information presented to them.

One of the tools available to researchers to implement eConsent is the eConsent Module [10] that is packaged with a Research Electronic Data Capture system called REDCap [11, 12]. This module has been available to REDCap users since 2018. This framework facilitates the documentation of the consent process, signing of the study/trial’s consent form by the participant and researcher in clinic, storing the consent form as a file for sites to download from within the REDCap instance, and sending the consent form to the participant via e-mail [10, 13]. The use of electronic systems to facilitate the documentation of the consent process is previously documented [5, 14–20] and appears to be the direction of travel for many academic clinical trials units (CTUs) within the UK [8].

In this article, we will build on the experiences of the Leeds [21] and Norwich CTUs [22] by describing the use of eConsent by studies in both the documentation of consent and in the delivery of consent materials managed by the Oxford Clinical Trials Research Unit (OCTRU), which is an academic CTU based in the UK, as are both Leeds and Norwich.

## Materials and methods

Within our academic CTU, we have utilised the REDCap eConsent module (Version 1.0) since 2019, using it on 35, including three COVID-19-related trials to date to document the consent process.

**Fig. 1:**
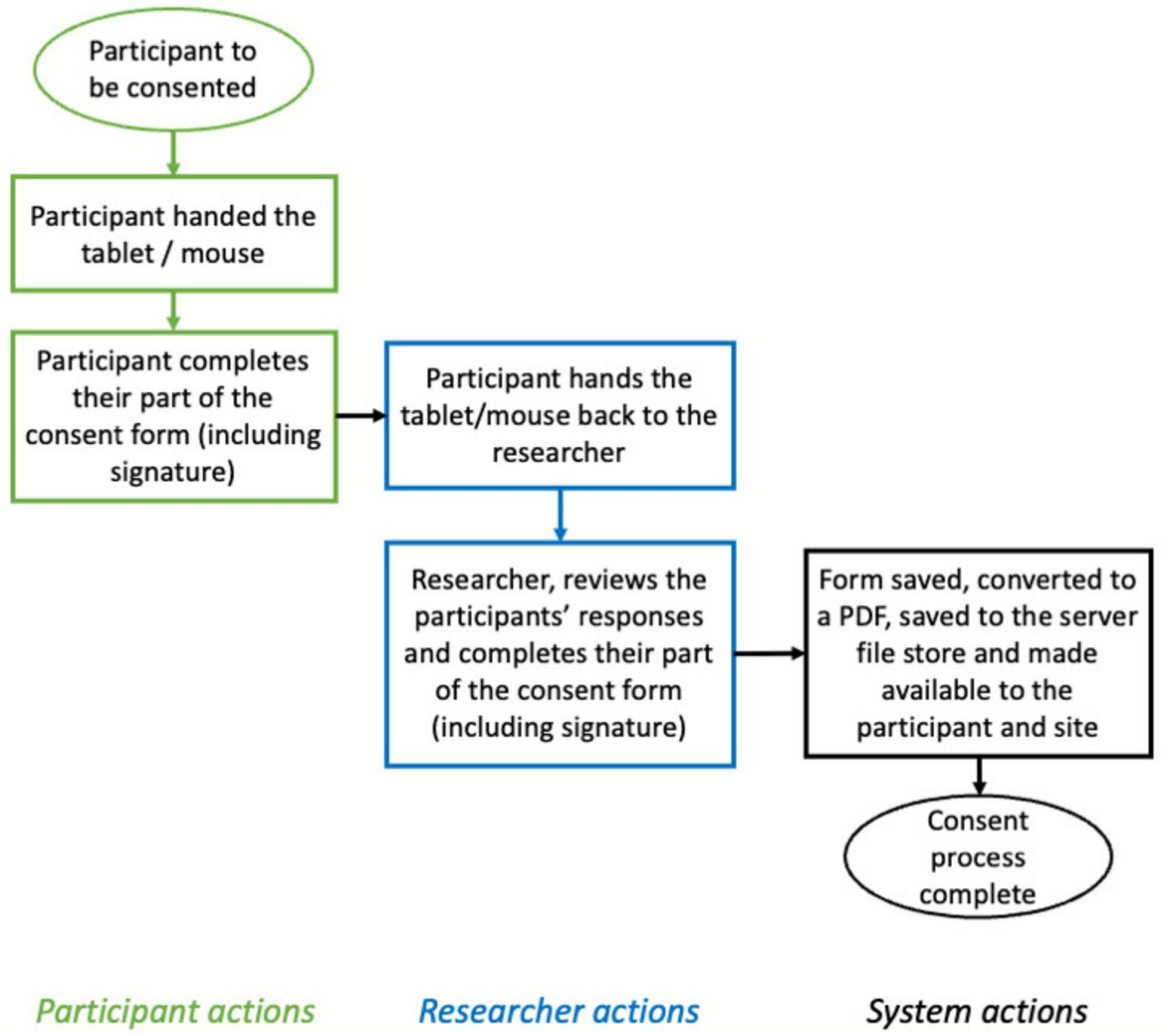
How documentation of eConsent takes place in clinical trials (in person)

Figure 1 shows how eConsent is used in general.

This process works well until the participant is not physically in clinic with the researcher; at this point, remote eConsent needs to be undertaken. REDCap does not immediately lend itself towards remote eConsent; as such, extensions (or external modules in REDCap parlance) to the underlying software had to be made to facilitate the process.

**Fig. 2:**
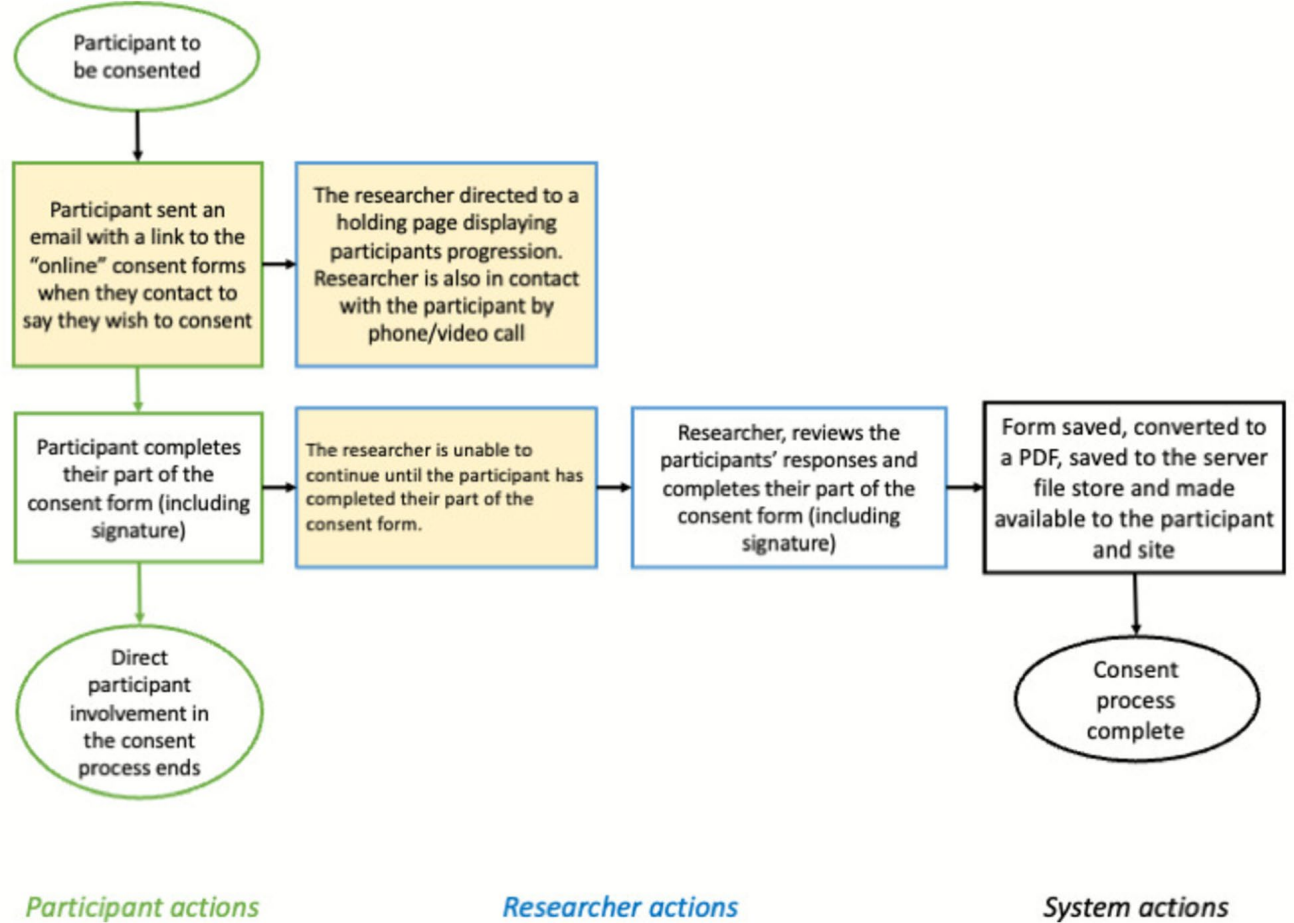
The remote eConsent process (the steps in yellow show the functionality added by our module)

**Fig. 3:**
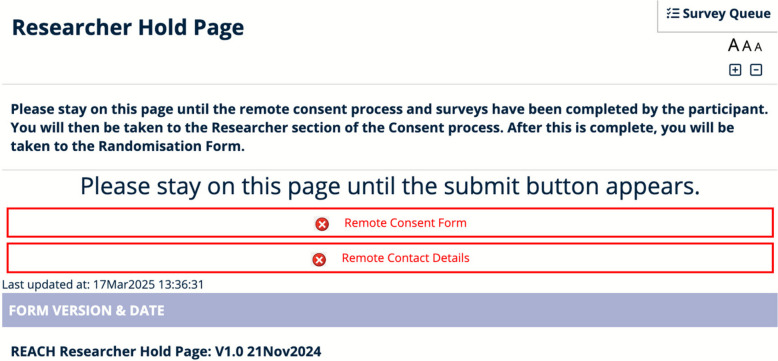
An example of the “Researcher Hold page”

The inbuilt REDCap eConsent module [10] works in the “Survey” mode of delivery; this does not require a unique login and is much more user-friendly to the end user. However, in some of our trials, data is collected through the “Forms” view, which is only available to authenticated (logged-in) users. The most useful parts of the REDCap eConsent module (the ability to send a copy of the consent form by e-mail to the nominated recipient and, importantly, to store the “original” PDF in a separate document library) are not available to those using “Forms” mode to document eConsent. To address this missing functionality, two external modules were written, tested, and implemented to facilitate the different approaches to the use of eConsent techniques in recording that the consent discussion has taken place. A “Forms-based eConsent Module” relies on the underlying eConsent module to be configured, even if “Survey” mode was not to be utilised. The second external module that was written was one to facilitate the remote eConsent process, in that a “Researcher Hold” page (see the process in Fig. 2) reloads on a regular basis, showing the participant’s progress. Figure 3 shows an example of the view that the researcher sees during this process.

Supplementary Document 1 lists the studies in our portfolio utilising eConsent since 2019 (as of 07 February 2025), and the properties of the studies (CTIMP/non-CTIMP, numbers of participants, method of consent, and availability of patient information material) are summarised in Table 1 (these data have been obtained from a direct review of the data collection systems and from the responses of individual trial managers to questions from the authors). In addition to participant consent, many studies also utilise parental consent/participant assent or facilitate consent from representatives. The REDCap module that we have written to facilitate this process can be configured for the specific needs of the study, as approved by the research ethics committee.

**Table 1:** Descriptors of the studies using eConsent and utilisation as of the time of writing

	**CTIMP studies**	**Non-CTIMP studies**	**Electronic consent methods not utilised**	**Non-CTIMP studies**
			**CTIMP studies**	
**Total studies**	5	30^a^	9	2
**Total participants consented**	861	11,556^b^	662	106
**Consent method utilised**				
In person (paper)	103	674	662	106
In person (electronic)	735	8909	N/A	N/A
Remote (verbal-phone)	0	269	N/A	N/A
Remote (electronic)	23	1706	N/A	N/A
**Patient information sheet available as (multiple options per study)** **follows**:				
Paper	5	29	7	2
Website	1	8	0	0
PDF	1	18	2	0
Explainer animation	1	18	0	0
Talking head	0	1	0	0

^a^Studies 10a and 10b in the supplementary material are counted as one study in this table, as are studies 23a and 23b. ^b^In one study, a participant was consented via two methods; both methods are accounted for in the method used, but the participant is only counted once in the totals

## Application of eConsent

Since 2019, we have utilised eConsent (always involving a participant and/or their representative and a delegated researcher) on 35 of our trials that have resulted in 12,417 individuals being consented, with 91% (11,309 of those being via an eConsent method, the remaining being consented via paper or telephone). In fact, we have only not programmed eConsent in 11 of our trials since 2019, and this was either because they were a single-centre trial, feasibility, or there was not time to program and test eConsent (for context since 2019, we have opened 46 new trials). Since 2019, we have created and utilised a remote eConsent pathway on 14 out of the 35 that we have set up.

As can be seen from Supplementary Document 1: Table 1, the most common part of eConsent that is utilized within our studies is that of documenting that the consent discussion has taken place, whilst the presentation of information in an electronic manner is used by half of our trials.

Whilst it is possible to decide about the use of eConsent in documenting the consent discussion at any point in a study’s lifecycle, decisions on the use of electronic methods to facilitate the consent discussion need to be made prior to submitting applications to funding bodies due to the need to account for design and programming time and the time needed to configure electronic tablets (such as “iPads”) coupled with potential costs associated with purchasing tablet devices, translations, the creation of animations, and the implementation of a standards compliant online presence.

**Fig. 4:**
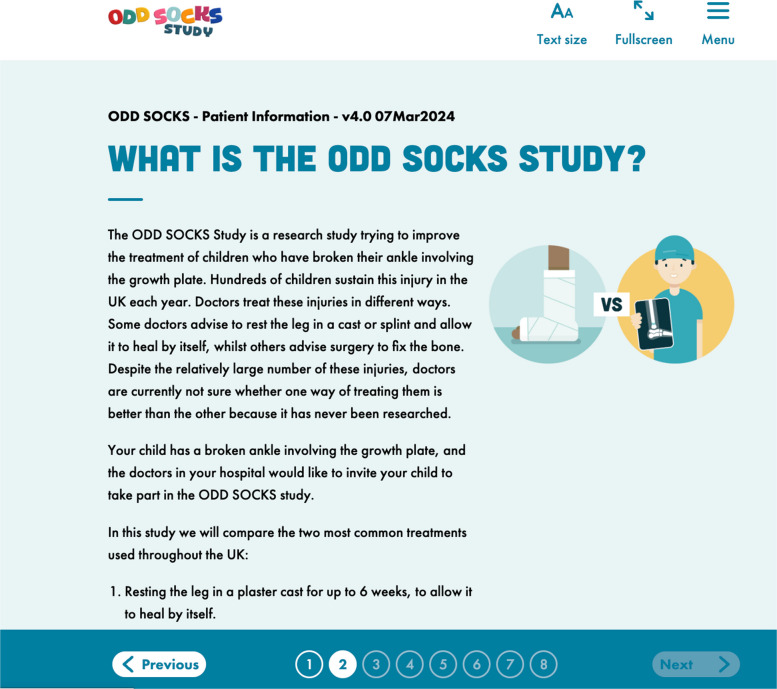
An example online patient information sheet, taken from the OddSOCKS study

As can be seen from Supplementary Document 1: Table 1, many of our studies also utilise eConsent to deliver the participant information material that is used to facilitate the informed consent discussion. Within our studies, generally, a specific participant information website is created (Fig. 4) to include all the content expected in a participant information sheet (PIS). This content is either displayed in a basic text format, as links to pdf documents, or via a series of pages (one or more per section) with the limitation that some pages cannot be accessed until previous pages have been viewed to the bottom of the page. In all cases, a PDF copy of the latest version of the PIS can be downloaded for offline access.

## Discussion

There is a pressing need for clinical trials to adapt to meet the demands of clinical practice and to meet the changing flow of patients through healthcare to maximise enrolment into clinical trials. The UK National Health Service (NHS) Long Term Plan in January 2019 set out goals of reducing face-to-face consultations by a third in 5 years through the creation of a more digitally enabled outpatient service [23]. Following the COVID-19 pandemic, there has been a large move away from face-to-face clinic visits, bringing savings for patients, the health service, and the environment, causing a paradigm shift in healthcare, with a huge emphasis on virtually accessed care [24, 25]. The UK Royal College of General Practitioners report that the way people access care has changed from 70% face to face and 30% remote prior to the pandemic, to 70% remote during the pandemic, and 50% remote post-pandemic [26]. This is the same in secondary care; even areas such as trauma and orthopaedics are seeing a huge growth in virtual follow-up [27].

This change in how some out-patient appointments are being delivered, alongside in trials where individuals are approached about participating in a trial and may then leave the hospital to take time to consider participating, has shown that the standard way of using eConsent did not work in these circumstances. Furthermore, the want from funders and participants to decentralise clinical trials (DCT) — so that the potential participant doesn’t necessarily come to the site/trial but the trial comes to the participant (or at least aspects of it), is spurring on the development of technologies and processes to facilitate this [28].

A review in 2024 highlighted that in moving towards embracing technology, there are four key constraints and considerations that must be addressed in moving towards employing successfully aspects of wholly decentralised clinical trials: participant demographics, participant motivation and incentives, the complexity of assigned tasks, and the clarity of scientific communication and instructions. The creation of ‘remote’ eConsent has set out to partially address one of these, but there are issues in the incorporation of this, including the dependence on technology and equipment requirements [29].

Most studies in our CTU portfolio use eConsent in one form or another, whilst in some the option for “paper” to be used is present, reflecting the needs of the patient population and of sites. Even if the study has been designed to employ eConsent, “paper” can and should always be an option for those with technology deprivation or technology phobias; otherwise, patients may be excluded from being able to decide if they wish to participate in a study, which is less than ideal and falls foul of the NIHR improving inclusion of underserved groups in clinical research (INCLUDE) [30]. It is important to note that most of our trial management groups (TMGs) have PPI representatives who have been very supportive of the expansion and use of eConsent — without this agreement we would not have incorporated eConsent into any of our trials. Furthermore, Supplementary Document 1 shows that eConsent can be utilised across all ages and multiple areas of medicine.

There are many advantages and disadvantages to the use of electronic forms for recording the consent discussion (these are discussed in more detail by De Sutter [31]): advantages include the ability to dynamically change font size in the web browser to make it easier to read (as shown at the top of Fig. 4), the ability to use screen readers (technology that reads the text content of a page), the ability to ensure that there are colour contrasts to facilitate easy readability, the reduction in paper usage, and the ability to include edit/validation checks on data entry to stop missing data or data entry issues. A common example being with paper consent forms, monitors will often find the entry of date of birth in the date of consent fields; today’s date can be auto inserted into an electronic form on signing. Apart from the “edit checks” (which aid in the reduction of the burden of trial monitors), these advantages also aid in the compliance with the UK Public Sector Bodies Accessibility Regulations 2018 [32] and the UK Equality Act 2010 [33]. The disadvantages in the use of these electronic methods include the following: a lengthier participant information sheet (although this is negligible if all data collection is electronic), an expectation of a level of familiarity with technology, and the potential need for additional electronic devices.

When utilising the electronic documentation of informed consent, decisions need to be made about which software should be employed, how the participant and clinician will interact with each other and with the software, how copies of the informed consent form can be provided to the participant and researcher, and then how any monitoring of the consent process can be enabled. In the trials reported here, the software utilised is REDCap [11, 12] with its associated eConsent module [10] as this is the software of choice within OCTRU for collecting all the trials’ data, including any electronic Patient reported Outcomes (ePRO’s). This being the case, one of the decisions made when applications for funding are made is “to provide iPads or not?”; frequently, the decision is that an iPad will be provided to each site not already involved in a related study. Whilst there are cheaper alternatives to iPads (e.g. Android tablets), anecdotal evidence from the FORCE study (in which half the sites were initially provided with iPads and the other half with Android tablets — ultimately replaced with iPads) [34] showed that research teams were more likely to be satisfied with an iPad rather than an Android device. One of the reasons for this preference was the lack of responsiveness from the Android devices. Where a tablet is sent to a recruiting site, it is configured and remotely managed by information technology (IT) staff at the University of Oxford. In some cases, the hospital’s Information Management & Technology (IM&T) teams request access to allow direct management of these iPads — this is generally governed by whether the iPad will connect to the hospital’s guest Wi-Fi network or will connect to the hospital’s protected Wi-Fi network. These iPads are generally returned to the central team for wiping and reconfiguration before being provisioned for reuse in other studies.

The advantage of using touchscreen devices/iPads is that both participants and researchers can make use of their fingers when digitally collecting a representation of their “wet” signatures, which is better than the use of a mouse to draw signatures. The disadvantage of using iPads is their cost, which is not insignificant for a large multicentre randomised controlled trial (RCT). Either way, the use of iPads facilitates compliance with the expectations of the Medicines for Human Use (Clinical Trials) Regulations 2004 [2] in the documentation of the informed consent process.

One of the challenges with eConsent is the proof of identity of the participant; in the studies reported here, proof of identity is obtained by asking for a confirmation of name and date of birth (which is standard NHS practice), either when talking to a patient face to face or at the start of any telephone/video call. To minimise the chance of a personal data breach under the UK General Data Protection Regulations (GDPR), when communicating electronically with participants (e-mail or text message), our processes have evolved to require e-mail addresses and telephone numbers to be re-entered (with an associated check that they are the same) and the inclusion of the ability to send a test message (either text or e-mail) to the contact details that have been recorded to confirm that the correct details have been entered (this has been achieved via the development of an external module for REDCap). One of the reasons that a signature is included on an informed consent form is to prove the identity of the completer. The types of signature that can be utilised on a consent form in trials within the United Kingdom (UK) are determined by the study risk [5, 35]; in all of the trials reported here, a simple electronic signature is defined as “that involve the participant tracing their handwritten signature using a finger or a stylus or biometric eSignatures should normally be used as these allow for direct comparison with eSignatures and/or wet-ink signatures previously used by the participant for the purpose of audit or where the consent is contested” [5, 36]. In the studies reported here, only “handwritten” signatures have been utilised when employing remote eConsent, as in the main the remote eConsent is being undertaken during a conversation with a healthcare professional; as such, identification is made in the standard way for the NHS.

## Things to consider

Building on the recommendations presented by Cragg et al. [21] and Hammond et al. [22], based on our experiences across all our studies, we recommend that future studies take the following into account when developing training, risk assessments, and processes for managing eConsent implementations.

1. Practicality for participants
  a aWithin REDCap, when collecting signatures, there is relatively little space for a signature to be drawn/provided; depending on the nature of their injury (e.g. wrist fractures or severe arthritis), this could cause issues for the participant (even if a tablet were utilised). Studies should ensure that they consider how they implement their eConsent forms from the perspective of the participant and the mechanisms used to collect “signatures”, e.g. providing for a hybrid approach or the ability for additional witnesses on the form.
  b How will the participant receive their copy of the consent form? By default, the REDCap system will store a copy in its file library but can also be configured to send a copy of the form to an e-mail address [10]. The choice of e-mail address should be appropriate to the setting in which consent is taken, e.g. directly to the participant or to the researcher to print out and hand to the participant.
  c Inclusivity for participants where English may not be their first language or who have poor eyesight.
2. Sites
  a Do sites need additional resources, such as electronic tablets, to utilise eConsent?
  b What extra training is needed and how will training in these processes be delivered?
  c Is Wi-Fi available in the areas of the hospital where the consent process takes place?
3. Quality assurance
  a Fraud detection: One of the advantages of using electronic systems such as REDCap is the inherent time stamps built into the data collection process. These time stamps, along with edit checks [37], can be utilised to ensure that the date of consent is as per that entered by the completers.
  b Data quality: Implementing these edit checks and constraints on data, such as date ranges, leads to higher quality and potentially fewer issues with consent forms.
  c Checks that the person consenting the participant is delegated to do so. In our CTU, we make use of electronic delegation logs, so we can automate checks of the consent form to ensure that those consenting are delegated to do so.
  d Data quality: Edit checks should be considered to provide feedback in real time to those completing the form, for example missing fields and is consent valid or not. Implementing such a strategy will aid in the quality of the forms being completed.
4. Information governance
  a Ensure that there is a process for removing participant e-mail addresses from the system should they ultimately decline to consent to participate in the study.
  b In the minority of cases where a consent form has been completed incorrectly (e.g. initials instead of names), what is the process that you will follow to make corrections and ensure that the participant has the updated copy of the form?
  c How to manage the storage of electronic images of a participant’s signature and how access to that signature is controlled and audited. For example, should the image files be retained once the consent form has been reviewed or should they be removed? Ensuring that you have guidance from your institutional information governance team or sponsor is important.

## Conclusions

We have successfully used eConsent techniques to receive informed consent from nearly 12,000 participants in a multitude of clinical trials. Half of these trials also used eConsent to facilitate both the documentation and the discussion around the consent process. Where remote eConsent has been used, techniques have been employed to make the process easier on the participant and the researcher, whilst not detracting from the two-way conversation that is important for the consent process.

### Limitations

We have reported upon details of the successful implementation of eConsent across a variety of studies; however, we have been unable to ascertain how many participants decided not to consent because of the electronic nature of the approach used.

## Supplementary Information

Supplementary Material 1: Table 1. eConsent can be utilised across all ages and multiple areas of medicine

## Abbreviations

CTU: Clinical trials unit
DCT: Decentralised clinical trial
eConsent: Electronic consent
ePRO: Electronic patient-reported outcomes
EDC: Electronic Data Capture
EME: Efficacy and mechanism evaluation
FDA: Food and Drug Administration
GCP: Good Clinical Practice
GDPR: UK General Data Protection Regulations
HRA: Health Research Authority
HTA: Health technology assessment
ICH: International Council for Harmonisation
IM&T: Information management & technology
INCLUDE: NIHR improving inclusion of underserved groups in clinical research
ISF: Investigator Site File
IT: Information technology
MHRA: Medicines and Healthcare products Regulatory Agency
NHS: National Health Service (UK)
NIHR: National Institute for Health and Care Research
OCTRU: Oxford Clinical Trials Research Unit
PDF: Portable document format
PIS: Participant information sheet
PPI: Patient and public involvement
RCT: Randomised controlled trial
RfPB: Research for patient benefit
REDCap: Research Electronic Data Capture
TMG: Trial Management Group
UK: United Kingdom
US: United States
Wi-Fi: Wireless Fidelity
