# Supplementary material for: Lessons learnt from the implementation of electronic consent (eConsent) and its use across a large portfolio of trials in a UK academic clinical trials unit
Source: Trials. 2026 Jan 23;27:75. doi: 10.1186/s13063-026-09445-5 (PMC12829000; doi:10.1186/s13063-026-09445-5)
Supplement: Supplementary file 1 — Supplementary Material 1: Table 1. eConsent can be utilised across all ages and multiple areas of medicine [file 13063_2026_9445_MOESM1_ESM.docx]

| **#** | **Brief description of the study/trial** | **CTIMP** | **Number consented to date** | **Consent methods used** | | | | **PIS availability** | **Comments** |
| --- | --- | --- | --- | --- | --- | --- | --- | --- | --- |
|  |  |  |  | **In person paper** | **In person electronic** | **Verbal (phone)** | **Remote electronic** |  |  |
| 1 | Phase 3 multi-centre, parallel-group, superiority individually randomised controlled study assessing the clinical effectiveness of supervised versus self –directed rehabilitation in improving ankle function for people aged 50 years and over after an ankle fracture. | No | 377 | No  (N/A) | Yes  (207) | Yes  (170) | No  (N/A) | Paper  Website  PDF  Explainer | Default documentation method was electronic.  Consent to be contacted about a qualitative study was added mid-way through the study.  Clinical staff consent to be contacted about the qualitative study was documented using paper forms. |
| 2 | Phase 2 multi-centre feasibility parallel-group, randomised double blind study to investigate the feasibility of a study comparing the impact of adalimumab injection versus placebo on pain and disease progression in the early painful phase of frozen shoulder. | Yes | 9 | Yes  (6) | Yes  (3) | No  (N/A) | No  (N/A) | Paper | Method for recording consent is determined by the site/participant at the point of consent. |
| 3 | Phase 3 multi-centre randomised superiority clinical trial of standard care and azithromycin versus standard care alone for adult patients with clinically diagnosed COVID-19 infection being managed on an ambulatory care pathway. | Yes | 298 | No  (N/A) | Yes  (298) | No  (N/A) | No  (N/A) | Paper  Website | Default documentation method was electronic. |
| 4 | Phase 3 multi-centre prospective randomised superiority trial of acute deformity correction versus pinning in situ for stable severe SCFE in children aged 8 years and above. | No | 15 | No  (N/A) | Yes  (14) | No  (N/A) | Yes  (1) | Paper  Website  PDF  Explainer | Default documentation method is electronic.  Also includes assent. |
| 5 | Phase 4 randomised trial comparing putting wrist fractures into a cast compared to putting the bones back into the correct position with metal plate or wires as necessary and then a cast. | No | 750 | No  (N/A) | Yes  (708) | No  (N/A) | Yes  (42) | Paper  Website  PDF  Explainer | Default documentation method was electronic. Not available via paper.  Also includes assent and consent to contact for participation in the qualitative study.  Remote eConsent added after six months.  Qualitative consent to contact was removed when the qualitative study had recruited sufficient participants. |
| 6 | Phase 3 multi-centre prospective randomised non-inferiority trial comparing a standard plaster cast versus a removable splint for patients aged 16 years and older with an acute fracture of the distal radius who do not require manipulation of the fracture. | No | 787 | No  (N/A) | Yes  (787) | No  (N/A) | No  (N/A) | Paper  Website  PDF  Explainer | Default documentation method is electronic. Not available via paper.  Also includes consent to contact for participation in the qualitative study – removed following the completion of the internal pilot. |
| 7 | Phase 3 randomised trial comparing a short vs long period of neo-adjuvant endocrine treatment before surgery for post-menopausal woman with breast cancer. | Yes | 158 | Yes  (97) | Yes  (38) | No  (N/A) | Yes  (23) | Paper  PDF  Explainer | Default documentation method is paper. |
| 8 | Phase 3 randomised trial comparing surgical fixation of severe ankle fractures compared to a non-surgical treatment – a close contact cast. | No | 891 | No  (N/A) | Yes  (803) | No  (N/A) | Yes  (88) | Paper  Website  PDF  Explainer | Default documentation method is electronic. Not available via paper.  Remote eConsent added post COVID. |
| 9 | Phase 3 prospective multi-centre randomised equivalence trial of a soft bandage with immediate discharge versus immobilisation with a splint for children with a torus fracture of the distal radius. | No | 965 | No  (N/A) | Yes  (965) | No  (N/A) | No  (N/A) | Paper  Explainer | Default documentation method is electronic. Not available via paper.  Also includes assent. |
| 10a | Pre study to ensure sites are able to definitely deliver a surgical trial intervention. | No | 71 | No  (N/A) | Yes  (8) | No  (N/A) | Yes  (63) | Paper  PDF | Default documentation method is electronic. Not available via paper. |
| 10b | Phase 3 prospective multi-centre definitive randomised controlled trial comparing the effects of using Diffusion Tensor Imaging (DTI) and intraoperative ultrasound (iUS) added to standard care technologies in the resection of glioblastomas. | No | 364 | No  N/A | Yes  (73) | No  N/A | Yes  (291) | Paper  PDF | Default documentation method is electronic.  System also facilitated proxy consent. |
| 11 | Phase 3 multicentre randomised trial comparing cast and brace treatment against surgery for those with a humeral shaft fracture. | No | 334 | No  (N/A) | Yes  (295) | No  (N/A) | Yes  (39) | Paper  Website  PDF  Explainer | Default documentation method was electronic.  Not available via paper. |
| 12 | Phase 3 multicentre randomised controlled trial examining the effects of temporarily pausing BTKi therapy to coincide with SARS-CoV-2 vaccinations and its impact on immune responses in patients with Chronic Lymphocytic Leukaemia. | No | 99 | Yes  (0) | Yes  (99) | No  (N/A) | No  (N/A) | Paper | Default documentation method was electronic.  Paper backups available at all sites. |
| 13 | Phase 3 prospective multicentre, two arm unblinded cluster randomised controlled trial of patients aged 60 and over who have had hip fracture surgery comparing standard of care mobilisation procedures and increased ward-based mobilisation activities. | No | 200 | No  (N/A) | Yes  (200) | Yes  (0) | No  (N/A) | Paper | Default documentation method is electronic.  Also includes all forms of consultee consent in England, Wales, Scotland and Norther Ireland, system determines the correct form to display to the researcher to be completed. |
| 14 | Phase 3 randomised trial comparing exploration and washout with microsurgical repair with exploration and washout alone for patients with digital nerve injuries | No | 251 | Yes  (226) | Yes  (25) | No  (N/A) | No  (N/A) | Paper | Default documentation method is paper.  Numbers were higher than sample size and not everyone consented was then suitable for randomisation. |
| 15 | Phase 3 multicentre prospective randomised superiority trial of conservative versus surgical treatments for displaced distal tibial fractures in children aged 8-15 years. | No | 29 | No  (N/A) | Yes  (27) | No  (N/A) | Yes  (2) | Paper  Website  PDF  Explainer | Default documentation method is electronic. Not available via paper.  Also includes assent and reconsent post-16. |
| 16 | Phase 3 prospective multi-centre trial comparing active versus surgical containment in children affected by Perthes’ disease. | No | 7 | No  (N/A) | Yes  (6) | No  (N/A) | Yes  (1) | Paper  Website  PDF  Explainer | Default documentation method is electronic. Not available via paper.  Also includes assent. |
| 17 | Phase 3 prospective multicentre trial comparing partial ablation of the prostate or radical treatment for men with localised intermediate-risk prostate cancer. | No | 64 | Yes  (35) | Yes  (1) | No  (N/A) | Yes  (28) | Paper | Default documentation method is paper. |
| 18 | Phase 3 prospective multicentre randomised trial of arthroscopic cleaning and tidying of partial tears of the rotator cuff tendon with and without a repair. | No | 33 | Yes  (33) | No  (N/A) | No  (N/A) | No  (N/A) | Paper  PDF | Study closed prior to implementation of eConsent. |
| 19 | Phase 3 prospective multicentre randomised trial of adults with a proximal humerus fracture comparing outpatient physiotherapy verses self-directly home-based exercise programmes. | No | 41 | No  (N/A) | Yes  (41) | No  (N/A) | Yes  (0) | Paper  PDF  Explainer | Default documentation method is electronic. Not available via paper. |
| 20 | Phase 3 prospective parallel group randomised controlled trial comparing an adolescent-specific strengthening programme versus usual care for ambulant adolescents with spastic cerebral palsy. | No | 66 | Yes  (6) | Yes  (60) | No  (N/A) | No  (N/A) | Paper  Website  PDF  Explainer | Default documentation method is electronic.  Also includes assent and reconsent post-16.  Awaiting implementation of remote eConsent to aid in workload management – requested by sites |
| 21 | Phase 3 prospective randomised multicentre open-label, two-arm parallel design, superiority Randomised Controlled Trial of intensive versus stand of care surveillance after treatment of oesophageal or gastric cancer. | No | 519 | No  (N/A) | Yes  (230) | No  (N/A) | Yes  (289) | Paper  PDF | Default documentation method is electronic. |
| 22 | Phase 3 prospective multicentre randomised controlled trial comparing treating epicondyle fractures in children. | No | 335 | Yes  (1) | Yes  (309) | Yes  (2) | Yes  (23) | Website  PDF  Explainer | Default documentation method is electronic. Not available via paper.  Also includes assent.  Remote eConsent added post COVID. |
| 23(a) | Phase 3 prospective randomised controlled trial comparing the addition of a skin flap onto the forearm of lung transplant recipients on receiving a transplant versus no flap – cohort consents. | No | 144 | Yes  (58) | Yes  (11) | No  (N/A) | Yes  (75) | Paper  Talking head | Default documentation is electronic. |
| 23(b) | Phase 3 prospective multicentre randomised controlled trial comparing the addition of a skin flap onto the forearm of lung transplant recipients on receiving a transplant versus no flap – cohort who go onto to be randomised. | No | 11 | Yes  (0) | No  (N/A) | Yes  (11) | No  (N/A) | Paper  Talking head | Default documentation is verbal. |
| 24 | Phase 3 prospective multicentre parallel group, randomised controlled trial of a child-specific dynamic stretching programme compared to usual care for children with spastic cerebral palsy. | No | 102 | Yes  (16) | Yes  (86) | No  (N/A) | No  (N/A) | Paper  PDF Explainer | Default documentation method is electronic.  Also includes assent and reconsent post-16.  Awaiting implementation of remote eConsent to aid in workload management – requested by sites |
| 25 | Phase 2 feasibility multicentre randomised controlled trial comparing autologous protein solution (APS) versus corticosteroids for treating subacromial shoulder pain. | No | 50 | No  (N/A) | Yes  (50) | No  (N/A) | No  (N/A) | Paper  Explainer | Default documentation method was electronic. |
| 26 | Phase 3 prospective multicentre randomised controlled trial comparing biopsy methods in men with suspected prostate cancer. | No | 1126 | Yes  (76) | Yes  (286) | No  (N/A) | Yes  (764) | Paper  PDF  Explainer | Default documentation method was electronic. |
| 27 | Pre-study to ensure sites are able to definitely deliver a surgical trial intervention. | No | 1 | Yes  (1) | Yes  (1) | No  (N/A) | No  (N/A) | Paper | Default documentation is electronic. |
| 28 | Phase 3 prospective multicentre randomised controlled trial of a continuation versus a temporary pause in Methotrexate treatment around the time of SARS CoV-2 vaccination in those with inflammatory conditions. | No | 425 | Yes  (11) | Yes  (414) | No  (N/A) | No  (N/A) | Paper  Explainer | Default documentation method was electronic.  One site used paper as they refused to use eConsent. |
| 29 | Phase 3 randomised trial comparing those allowed to weight bear at 2 weeks following surgery for an unstable ankle fracture compared to those waiting 6 weeks before weight bearing. | No | 562 | No  (N/A) | Yes  (562) | No  (N/A) | No  (N/A) | Paper  PDF  Explainer | Default documentation method was electronic.  Implemented remote eConsent following covid |
| 30 | Phase 3 prospective multicentre randomised controlled trial comparing in adults with a hip fracture the effect of an infusion of lidocaine during surgery on delirium symptoms. | Yes | 379 | Yes  (0) | Yes  (379) | Yes  (0) | No  (N/A) | Paper | Default documentation method is electronic.  Paper and verbal consent only allowed in exceptional circumstances.  Also includes all forms of consultee consent in England, Wales, Scotland and Norther Ireland, system determines the correct form to display to the researcher to be completed. |
| 31 | Phase 3 prospective multicentre randomised controlled superiority trial comparing fixing broken hip bones versus performing a hip replacement. | No | 643 | Yes  (2) | Yes  (640) | Yes  (1) | No  (N/A) | Paper | Default documentation method is electronic.  Paper and verbal consent only allowed in exceptional circumstances.  Also includes all forms of consultee consent in England, Wales, Scotland and Norther Ireland, system determines the correct form to display to the researcher to be completed. |
| 32 | Phase 3 prospective multicentre randomised controlled superiority trial comparing a dual mobility total hip replacement to standard total hip replacement. | No | 222 | Yes  (0) | Yes  (222) | Yes  (0) | No  (N/A) | Paper | Default documentation method is electronic.  Paper and verbal consent only allowed in exceptional circumstances.  Also includes all forms of consultee consent in England, Wales, Scotland and Norther Ireland, system determines the correct form to display to the researcher to be completed. |
| 33 | Phase 3 prospective multicentre randomised controlled trial comparing in adults with a hip fracture the effect of an infusion of infliximab during surgery on delirium symptoms. | Yes | 17 | Yes  (0) | Yes  (17) | Yes  (0) | No  (N/A) | Paper | Default documentation method is electronic.  Paper and verbal consent only allowed in exceptional circumstances.  Also includes all forms of consultee consent in England, Wales, Scotland and Norther Ireland, system determines the correct form to display to the researcher to be completed. |
| 34 | Phase 4 prospective multicentre randomised controlled trial comparing adults post hip fracture using off-loading devices, low pressure devices and usual care whilst laid in bed. | No | 1443 | Yes  (2) | Yes  (1441) | Yes  (0) | No  (N/A) | Paper | Default documentation method is electronic.  Paper and verbal consent only allowed in exceptional circumstances.  Also includes all forms of consultee consent in England, Wales, Scotland and Norther Ireland, system determines the correct form to display to the researcher to be completed. |
| 35 | Phase 3 randomised trial comparing progressive resistance and flexibility exercises versus usual care advice after a distal radius fracture in adults aged 50 or over. | No | 629 | No  (N/A) | Yes  (374) | Yes  (255) | No  (N/A) | Paper  PDF  Explainer | Default documentation method was electronic. |
